# Professor Means

**Published:** 1858-03

**Authors:** 


### Professor Means.

As in the January number of this Journal, we announced that our friend, Prof. ALEXANDER MEANS, had *resigned* the Chair of Chemistry and Pharmacy in the Atlanta Medical College, we now state that Professor Means has subsequently *withdrawn* that resignation, and, therefore, retains his connection with that Institution.

Professor MEANS has since resigned the Chair of Chemistry and Pharmacy, in the Medical College of Georgia.—*Augusta Medical Journal.*

☞ From the above announcement, it will be perceived that the *Atlanta Medical College*, is now in *exclusive* possession of the services of the eminent gentleman referred to.

